# Nonuniform sliding-window reconstruction for accelerated dual contrast agent quantification with MR fingerprinting

**DOI:** 10.1007/s10334-023-01140-9

**Published:** 2024-01-13

**Authors:** Anna Marriott, James Rioux, Kimberly Brewer

**Affiliations:** 1 Biomedical MRI Research Laboratory (BMRL), IWK Health Centre, 5850/5980 University Avenue, Halifax, NS B3K 6R8 Canada; 2 Department of Physics and Atmospheric Science, Dalhousie University, Halifax, NS Canada; 3 Biomedical Translational Imaging Centre (BIOTIC), NS Health, Halifax, NS Canada; 4 Department of Diagnostic Radiology, Dalhousie University, Halifax, NS Canada; 5 School of Biomedical Engineering, Dalhousie University, Halifax, NS Canada; 6 Department of Microbiology and Immunology, Dalhousie University, Halifax, NS Canada

**Keywords:** MR fingerprinting, *T*_2_*, Sliding window, Iron, Quantitative MRI, Preclinical

## Abstract

**Objective:**

MR fingerprinting (MRF) can enable preclinical studies of cell tracking by quantifying multiple contrast agents simultaneously, but faster scan times are required for in vivo applications. Sliding window (SW)-MRF is one option for accelerating MRF, but standard implementations are not sufficient to preserve the accuracy of *T*_2_*, which is critical for tracking iron-labelled cells in vivo.

**Purpose:**

To develop a SW approach to MRF which preserves the *T*_2_* accuracy required for accelerated concentration mapping of iron-labelled cells on single-channel preclinical systems.

**Methods:**

A nonuniform SW was applied to the MRF sequence and dictionary. Segments of the sequence most sensitive to *T*_2_* were subject to a shorter window length, preserving the *T*_2_* sensitivity. Phantoms containing iron-labelled CD8+ T cells and gadolinium were used to compare 24× undersampled uniform and nonuniform SW-MRF parameter maps. Dual concentration maps were generated for both uniform and nonuniform MRF and compared.

**Results:**

Lin’s concordance correlation coefficient, compared to gold standard parameter values, was much greater for nonuniform SW-MRF than for uniform SW-MRF. A Wilcoxon signed-rank test showed no significant difference between nonuniform SW-MRF and gold standards. Nonuniform SW-MRF outperformed the uniform SW-MRF concentration maps for all parameters, providing a balance between *T*_2_* sensitivity of short window lengths, and SNR of longer window lengths.

**Conclusions:**

Nonuniform SW-MRF improves the accuracy of matching compared to uniform SW-MRF, allowing higher accelerated concentration mapping for preclinical systems.

## Introduction

Contrast agents are routinely used to enhance the ability of MRI to differentiate between healthy and unhealthy tissue, allowing the detection of pathological changes with high sensitivity, specificity, resolution and penetration [1]. This enables longitudinal in vivo studies to collect detailed information on migration patterns of contrast agents, which strengthens molecular imaging studies, particularly those using cell tracking.

MR cellular imaging is already crucial for gathering information on immune cell subsets, and has been used by several groups to follow adoptive transfer of immune cells that are used as a therapy or to track immune cells in disease models, both untreated and treated with immunotherapy agents [2–6]. Whilst cellular MRI is most commonly performed with iron-based contrast agents due to their high relaxivity, biocompatibility and ease of cellular labelling [7], contrast agents targeting specific molecular species are becoming more popular to probe a wider range of cellular and molecular targets [3, 4, 8, 9]. Unfortunately, current MR cell tracking techniques are limited to only a single contrast agent tracked at once, greatly limiting the complexity or speed of preclinical studies. Further improvements in efficacy, quantification capabilities, scan times, and financial costs are critical for developing MR cell tracking. Access to simultaneous tracking of two contrast agents has the potential to address these needs.

Magnetic Resonance Fingerprinting (MRF) has been shown to meet the criteria for simultaneous contrast agent imaging [10]. MRF relies on a novel acquisition strategy which provides multiple relaxation maps simultaneously and is highly robust to undersampling, leading to greatly accelerated acquisition times. Previously, it was demonstrated that MRF can provide concentration maps for multiple contrast agents simultaneously, referred to as dual contrast MRF [11]. This was expanded to include *T*_2_* contrast agents, a critical step in adapting MRF for tracking of iron-labelled cells in preclinical MRI [12]. Whilst that work demonstrated the potential for in vivo dual contrast MRF including *T*_2_*, it required a scan time of over 400 s per slice, which is far too slow to capture the temporal dynamics of many contrast agents, making it logistically unfeasible to use.

A number of traditional and state-of-the-art techniques can be used for MRF acceleration, but many are not accessible in a preclinical setting. For example, a lack of a multichannel RF coil inhibits the use of compressed sensing, and insufficiently sized datasets preclude machine learning-assisted acceleration [13, 14]. In vivo dual contrast MRF for use in cell tracking requires additional technical development to produce the required resolution and accuracy in a preclinical setting.

Some techniques remain applicable to preclinical MRF for accelerated acquisition, including novel parameter regimes to lessen the total length of the sequence [15], and 3D acquisition to utilise undersampling in the Z plane [16]. One method of note is the sliding window (SW) reconstruction. Originally proposed for dynamic contrast-enhanced imaging, SW reconstruction offers a method of trading temporal fidelity for improved signal-to-noise ratio (SNR) by combining undersampled subsets of data to produce less undersampled images [17]. SW reconstruction has already been used in MRF studies, and has demonstrated the ability to improve the image quality of reconstructed frames, leading to more accurate fingerprint matching [18] and allowing for motion suppression [19]. However, complications arise when applying this technique to *T*_2_* sensitive methods. Short window lengths fail to provide enough SNR to highly undersampled MRF preclinical datasets, whilst application of longer window lengths can lead to greater degradation in the *T*_2_* sensitivity, necessitating additional care when a SW is applied to preclinical multiparametric MRF.

If MRF is to be fully utilised for MR immune cell tracking, it is vital that acceleration techniques available for a preclinical setting, such as SW, are further improved to include accurate *T*_2_* quantification. This would allow dual contrast cellular imaging, including an iron-based contrast agent, on an in vivo appropriate timescale. We aim to address this issue by implementing a ‘nonuniform’ SW to provide higher temporal resolution in the regions of the sequence which are most sensitive to *T*_2_* changes, enabling higher accelerated quantification of two contrast agents simultaneously, within 80 s per slice.

## Materials and methods

### Pulse sequence design

In this work, a non-balanced steady-state free precession (SSFP) sequence is the basis for the MRF acquisition and dictionary, similar to previous implementations of *T*_2_* MRF [12]. An adiabatic inversion pulse is applied at the beginning of the sequence to improve *T*_1_ sensitivity, followed by 1000 imaging frames of varying TR, TE and flip angle (FA) (TBW = 8). Three distinct segments of parameter variation are employed to enable *T*_2_* sensitivity. In the first segment, TR, TE and FA are held constant, to create a traditional steady-state free precession decay, for a total of 200 imaging frames. The second segment of 400 imaging frames contains Perlin noise varied TR (between 16 and 20 ms) and FA (between 20 and 60°), whilst TE is held constant at 2 ms. The final section contains a stationary 30° FA, whilst TE is varied sinusoidally between 5 and 10 ms. Whilst it has been shown that TR variation may be unnecessary for MRF [20], this final section contains TR variation to allow sufficient room for TE variation in the shortest possible acquisition time.

The trajectory for *k*-space acquisition is a variable density spiral, which fully samples the centre of *k*-space with 8 interleaves, and the outer region fully within 64 interleaves. Each interleaf samples 923 points with a dwell time of 5 μs. The maximum slew rate and gradient strength are set to 200 T/m/s and 91 mT/m respectively, corresponding to ~ 66% of the hardware maximum. Dephasing of at least 2 pi radians is achieved using a 3 ms crusher gradient between each set of 1000 imaging frames, followed by a 0.5s delay to relax the spins into thermal equilibrium. Total imaging time per slice is approximately 30 s, consisting of 1000 imaging frames with 2 interleaves collected for each frame. Interleaves are rotated by the golden angle between each imaging frame, as well as between each set of 1000 imaging frames. The resulting MRF images have an FOV of 35 mm^2^, with a slice thickness of 1 mm, and a matrix size of 64 × 64.

### Sliding window reconstruction

In our previous work, we demonstrated that single-channel MRF with *T*_2_* sensitivity achieved sufficient image quality for in vivo application up to 4× undersampling. For in vivo MRF to provide multi-slice imaging in an in vivo appropriate timeframe, this must be accelerated to 24x, whilst providing sufficient data fidelity to still enable dual contrast agent mapping. Preliminary tests suggested that the application of a SW reconstruction improved SNR, but hindered *T*_2_* quantification.

**Fig. 1:**
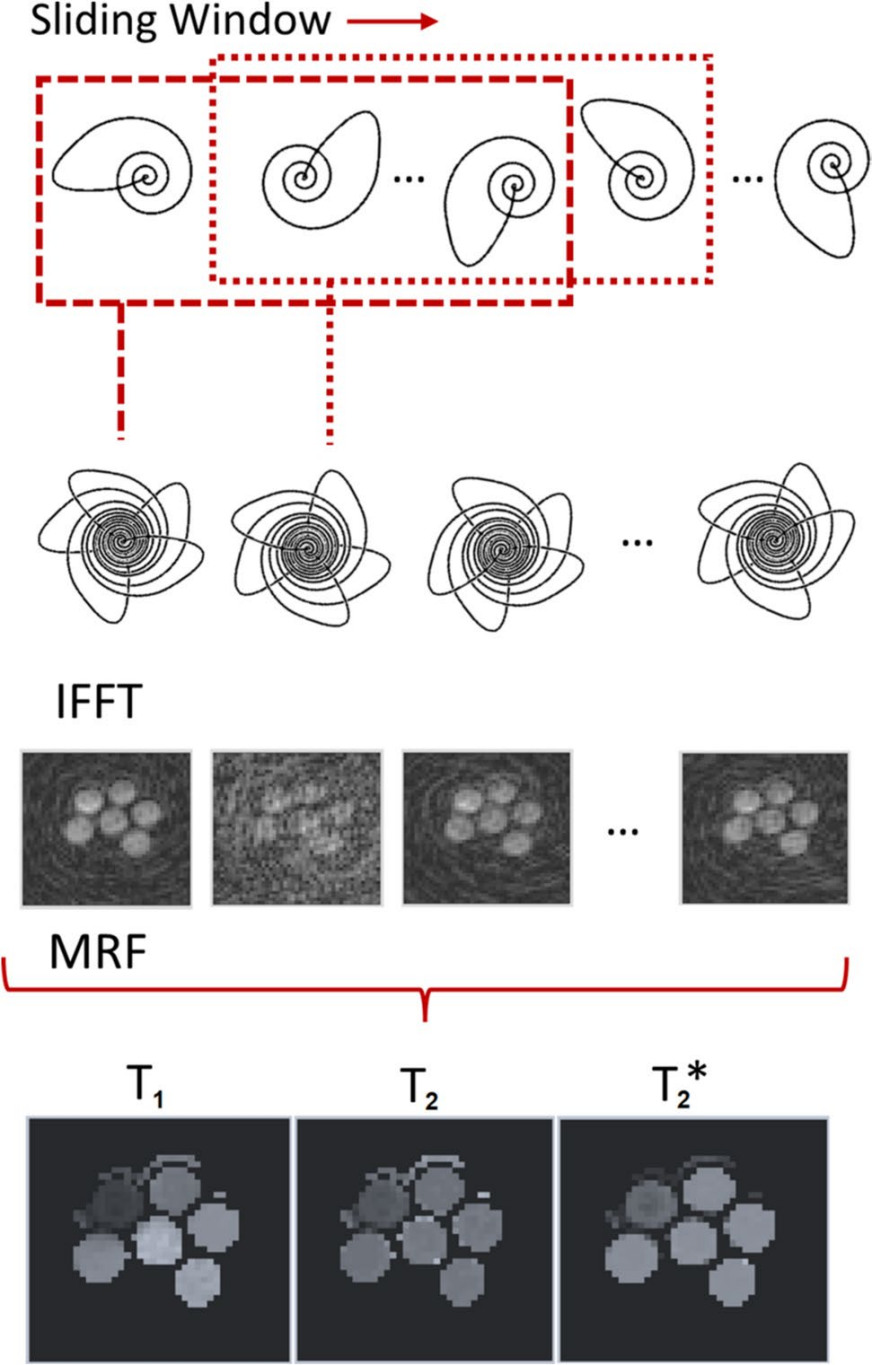
Workflow of Sliding Window Reconstruction. Prior to reconstruction via IFFT, individual interleaves are combined. This trades temporal resolution for increased spatial fidelity. When applied to MRF, the same sliding window reconstruction must also be applied to the dictionary

Sliding window reconstruction for MRF is outlined in Fig. 1. Prior to inverse nonuniform fast Fourier transform (NUFFT), the *k*-space data from the individual timeframes are combined to produce a new set of *k*-space data. After reconstruction, each image now represents a wider temporal footprint (and therefore decreased temporal fidelity) but has increased SNR and fewer undersampling artefacts.

**Fig. 2:**
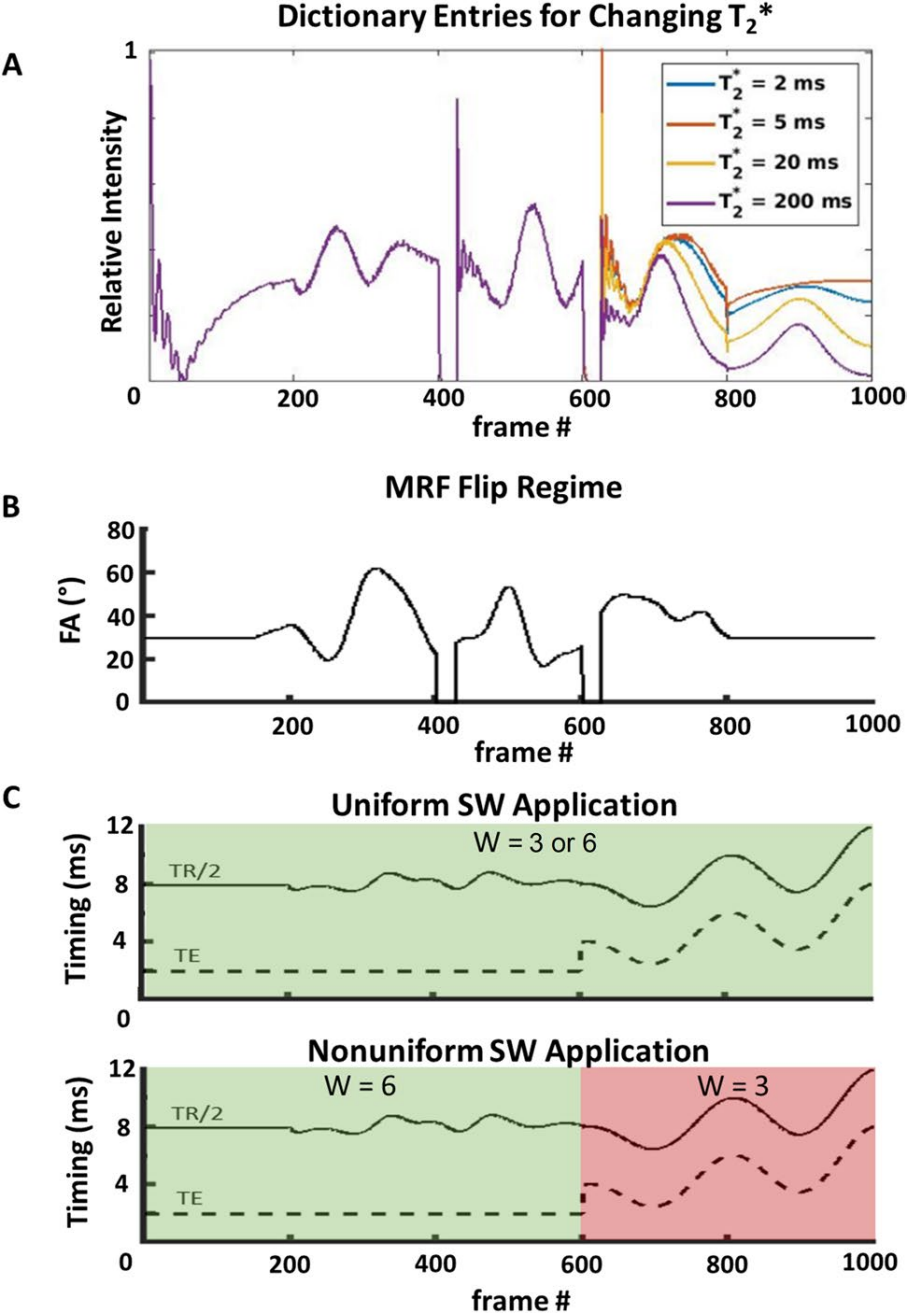
**A** Dictionary entries for fingerprints with different *T*_2_* values. *T*_2_* sensitivity is confined to the final 400 frames of the MRF sequence. **B** Flip angle used for each imaging frame of MRF. **C** Uniform and nonuniform SW application. Uniform SW application is required to allow for sufficient undersampling of *T*_2_* MRF to reach preclinically accepted scan times, but impedes *T*_2_* accuracy at long window length. In nonuniform SW, the final 400 frames (red) are subject to a shorter sliding window length of 3, preserving *T*_2_* sensitivity whilst still allowing for higher acceleration factors

The proposed nonuniform SW seeks to optimise MRF to allow both a higher undersampling factor and *T*_2_* sensitivity, for use on a single-channel RF coil. This is achieved by maintaining a shorter SW length during the parts of the MR fingerprint which are most sensitive to *T*_2_*. Figure 2a shows the fingerprints of the proposed sequence for a range of *T*_2_* values, where the final 400 frames of TE modulation encapsulate the *T*_2_* sensitivity. The proposed method applies a sliding-window similar to previous MRF studies [18], combining a fixed number of consecutive interleaves to generate higher-quality images. The length of this window is decreased over the final 400 imaging frames to create a novel, nonuniform SW, trading potential image quality for higher *T*_2_* sensitivity. This is visually demonstrated in Fig. 2c. For this work, nonuniform SW refers to a window width *W* of 6 for the initial 600 frames, and *W* = 3 for the final 400 frames. This is compared to two different uniform window lengths, *W* = 3, and *W* = 6. These window lengths were chosen to demonstrate nonuniform SW’s ability to provide a compromise between the high acceleration of long window length, and the *T*_2_* sensitivity of short window length.

### Dictionary design and parameter estimation

The dictionary is based on the extended phase graphing (EPG) framework [21] simulating SSFP sequences without concern for the off-resonance component which would be present in a balanced sequence. Repetition frames were included at the beginning of dictionary simulation to create a steady-state dictionary response, to account for the short delay between individual interleaf acquisition [15]. *T*_2_* decay is simulated by scaling signal according to the echo time at each imaging frame by exp(− TE/*T*_2_*). The dictionary entries range in value from *T*_1_ = 10–4000 ms, *T*_2_ = 5–1000 ms, and *T*_2_* = 1–250 ms, all with an increment of 5% per step. Illogical entries were removed, such as entries where *T*_1_ < *T*_2_.

After dictionary generation of ~ 600,000 entries taking roughly 15 min, a sliding window is applied to all entries. For uniform SW, *W* = 3, or *W* = 6 was applied to all 1000 frames. For nonuniform SW, *W* = 6 was used for the first 600 entries in a fingerprint and *W* = 3 for the final 400 imaging frames. Since the dictionaries are sparse, they can be subject to singular value decomposition (SVD) without negatively impacting matching capability, as is regularly implemented to reduce the size of MRF dictionaries [22]. With a rank of 25, SVD compresses the dictionary to 2.5% of its original size. Sliding window and SVD dictionary processing take approximately 1 h. Reconstruction and matching were performed in MATLAB (The MathWorks; Natick, MA), on a virtual machine with 200GB memory and 24 CPUs running on a Linux enterprise cluster. *T*_1_, *T*_2_ and *T*_2_* maps are generated using a traditional MRF pipeline [10], matching the maximum dot product between a voxel and the dictionary.

### Phantom evaluations

All experiments were performed on a 3T preclinical system (Agilent, Santa Clara, CA), with a quadrature RF coil. Phantoms consisting of 8% gelatin and CD8+ T cells labelled with a range of ProHance (Gadoteridol; Bracco Imaging) and/or Molday ION™ Rhodamine B SPIO (BioPal Inc., Worchester, Massachusetts, USA) were prepared in 5 mm NMR tubes, with concentrations chosen to mimic the parameter range of expected future in vivo scans. These concentrations can be found in Table 1. CD8+ T cell isolation was done using the same procedure as in previous *T*_2_* MRF literature [12]. Gold standard *T*_1_, *T*_2_ and *T*_2_* values were obtained to validate the proposed modifications to SW-MRF. *T*_1_ values were obtained using a multi-TI inversion recovery sequence (TI = 0.05, 0.01, 0.02, 0.4, 0.8, 1.6, 3.2 s, 64 × 64, 1 mm slice thickness). *T*_2_ was generated using a CPMG sequence, with ETL = 10, echo spacing = 33 ms (64 × 64, 1 mm slice thickness). *T*_2_* values were obtained by measuring the linewidth of individual samples with a non-spatially resolved hard pulse and using the relation *T*_2_* = 1/(*π *× linewidth). Averages of the centre 3 × 3 voxels of the MRF parameter maps were measured and compared to the gold standard values using Lin’s concordance correlation coefficient (CCC) in order to express a quantifiable representation of the deviation from the gold standard for uniform sliding window lengths, and for the proposed nonuniform sliding window approach. Wilcoxon signed-rank tests were used to examine the distributions of the relaxation parameters computed with SW-MRF and those calculated from the gold standard measurements, with *p* < 0.05 representing a significant difference in the means of the distributions.

**Table 1:** Composition of the phantoms used in the study. Gold standard parameter measurements are included for each phantom

I											
Phantom	1	2	3	4	5	6	7	8	9	10	11
Gad (mM)	0.45	0.4	0.35	0.3	0.2	0.1	0	0.2	0	0.2	0.4
cells/mL (× 10^6^)	0.125	0.25	0.5	1.25	3.75	6.25	0	1.25	1.25	7.5	7.5
*T*_1_ (ms)	299	312	357	416	474	551	1514	523	1287	393	271
*T*_2_ (ms)	259	270	254	212	113	78	1074	214	282	79	71
*T*_2_* (ms)	140	105	109	96	104	74	230	95	102	78	70

MRF parameter maps were used to generate dual concentration maps for both contrast agents, via an expansion to the linear relaxation model [11, 12]

1 $$R_1 = R_{1,0} + r_{1A} \times [A] + r_{1B} \times \left[ B \right]$$

2 $$R_2^* = R_{2,0}^* + r_{2A}^* \times [A] + r_{2B}^* \times [B]$$

where *R*_1_/*R*_2_* are the reciprocal of *T*_1_/*T*_2_*, *R*_1.0_/*R*_2.0_* are the values of *R*_1_/*R*_2_* without contrast added, measured from phantom 7 using MRF. [*A*] and [*B*] are the concentrations of iron-labelled cells or ProHance, *r*_1A_ and r*_2A_ are the magnetic relaxivities of the iron-labelled cells, and *r*_1B_ and r*_2B_ are the magnetic relaxivities of ProHance. Calculation of these values is provided in the supplementary material of our previous publication [12]. This was performed for both a uniform window length of 6, and the proposed nonuniform window length. 3 × 3 voxel averages of the MRF-produced concentration maps were extracted, with CCC used to evaluate the performance of both uniform and nonuniform SW-MRF with respect to the known concentration values of contrast agents.

## Results

### Parameter validation

**Fig. 3:**
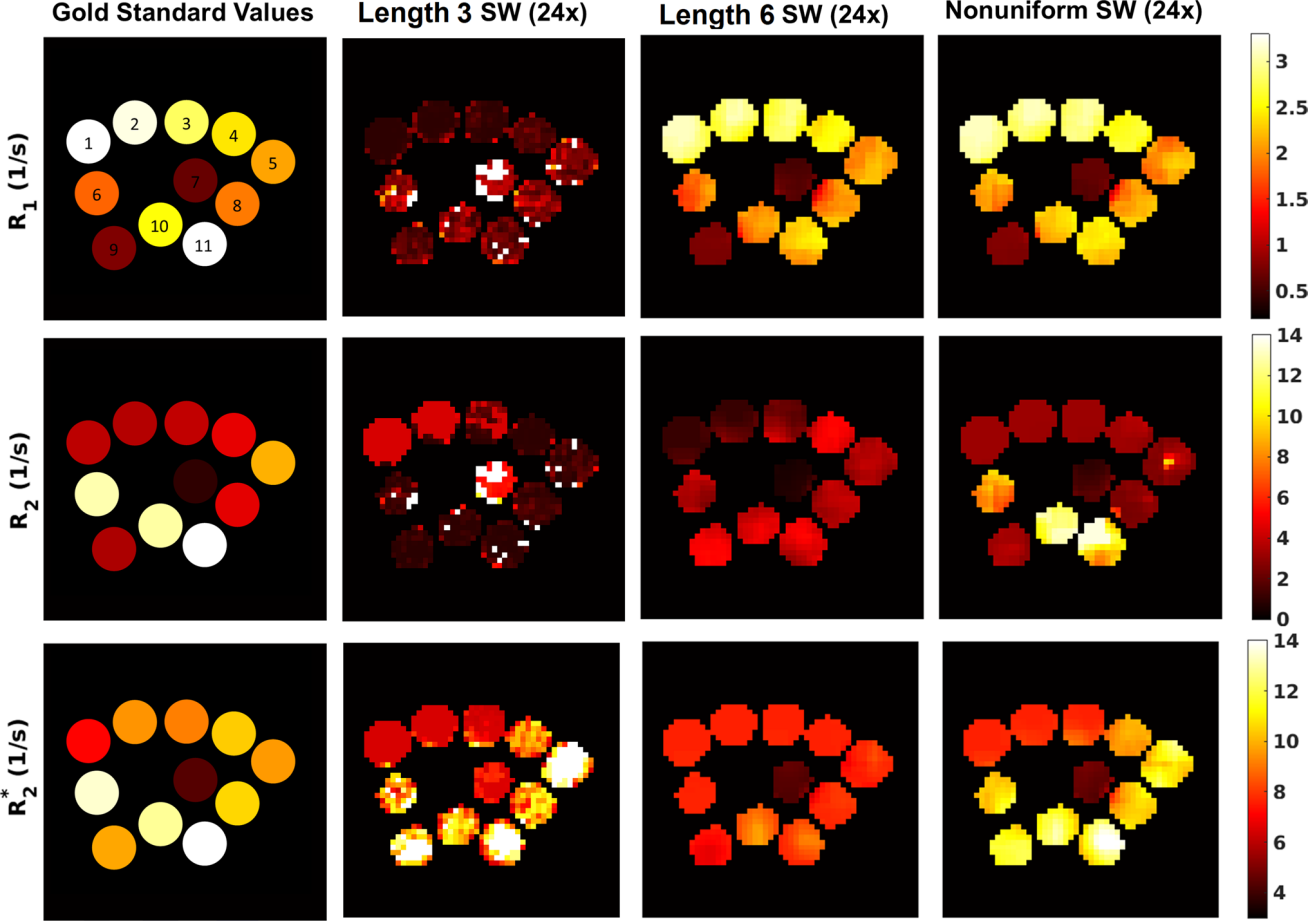
Comparison between gold standard parameter values (far left column), 24× undersampled uniform SW for a window length of either 3, 6 (middle columns), or 24× undersampled nonuniform SW (far right column) MRF. Phantom numbers refer to Table 1 for the composition and gold standard parameter values of each phantom

Figure 3 shows the resulting relaxation rate maps for highly undersampled SW-MRF with different SW regimes, compared to known values. Here the relaxation rates are mapped, to provide a better visualisation of the dynamic range present. Visual inspection of the parameter maps for the uniform SW-MRFs shows that parameter variation is not accurately captured when compared to that of the known values, specifically for *R*_1_ and *R*_2_ for the shorter uniform window length, and *R*_2_ and *R*_2_* for a longer uniform window length. Conversely, nonuniform SW-MRF demonstrates parameter changes between phantoms much closer to that seen in the gold standard. Overall, a sliding window length of 6 leads to a less noisy parameter map but hinders *R*_2_* sensitivity.

**Fig. 4:**
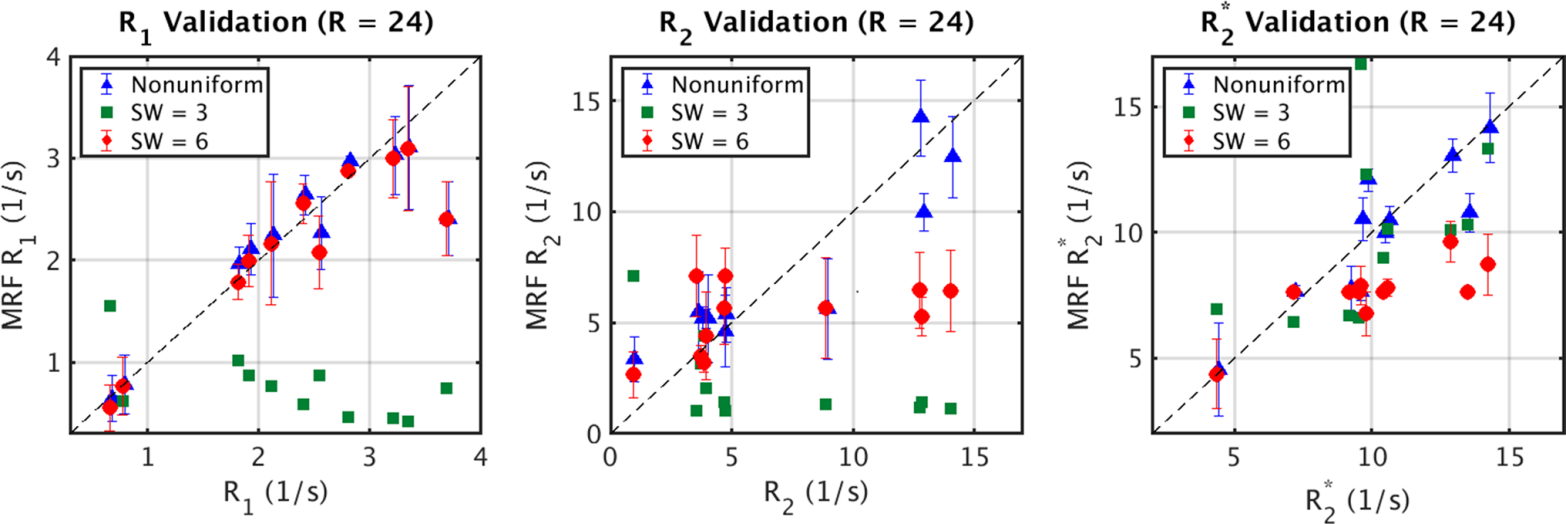
Comparison of the gold standard values for each phantom, compared with the average over a 3 × 3 voxel ROI taken from the nonuniform SW-MRF (triangle), uniform SW = 6 MRF (circle), and uniform SW = 3 MRF (square). Note that the SW = 3 error bars were very large due to the increased noise, and were removed for clarity

Figure 4 shows the comparison between the gold standard parameter values of each phantom to the 3 × 3 voxel average of the uniform and nonuniform SW-MRF. For *R*_1_, SW = 3 fails to capture the correct values, but both the SW = 6 and nonuniform SW perform well, with almost every phantom matching the gold standard *R*_1_ values. The one exception is phantom 11, which contains the highest concentration of both contrast agents, and therefore the highest *R*_1_ value. For *R*_2_ and *R*_2_*, the SW = 6 fails to capture the full dynamic range required for accurately mapping the phantoms with higher concentrations, in both cases underestimating the phantoms with *R*_2_/*R*_2_* values of 10 s^−1^. SW = 3 has similar difficulties quantifying *R*_2_, but outperforms a longer SW length for evaluating *R*_2_*, despite producing parameter maps with more noise. Nonuniform SW outperforms both uniform sliding window lengths for both *R*_2_ and *R*_2_*. Table 2(a) shows the CCC values associated with Fig. 4 which align with these findings. Table 2(b) shows the Wilcoxon signed-rank test results between the uniform and nonuniform SW results from Fig. 4, where nonuniform SW parameters are shown to not significantly differ from the gold standard, unlike SW = 6 *R*_2_* and SW = 3 *R*_1_/*R*_2_, where *p* < 0.05.

**Table 2:** (a) Lin’s CCC values for Fig. 4, (b) Wilcoxon signed-rank test results between the gold standard parameter values, and uniform/nonuniform phantom data in Fig. 4, where *p* < 0.05 represents a significant difference in the distributions, (c) Lin’s CCC values for the agent concentration data in Fig. 5b

(a)			
Lin’s CCC	*R*_1_	*R*_2_	*R*_2_*
SW = 3	− 0.1115	− 0.2181	0.6669
SW = 6	0.8801	0.2723	0.3482
Nonuniform SW	0.8803	0.8837	0.8776

(b)			
Wilcoxon signed-rank test	*R*_1_	*R*_2_	*R*_2_*
SW = 3	0.0047	0.0322	0.6301
SW = 6	0.2402	0.5195	0.0049
Nonuniform SW	0.7002	0.7646	0.8311

(c)		
Lin’s CCC	Gad Conc	Cell Conc
SW = 6	0.902	0.4666
Nonuniform SW	0.8874	0.8226

### Concentration validation

**Fig. 5:**
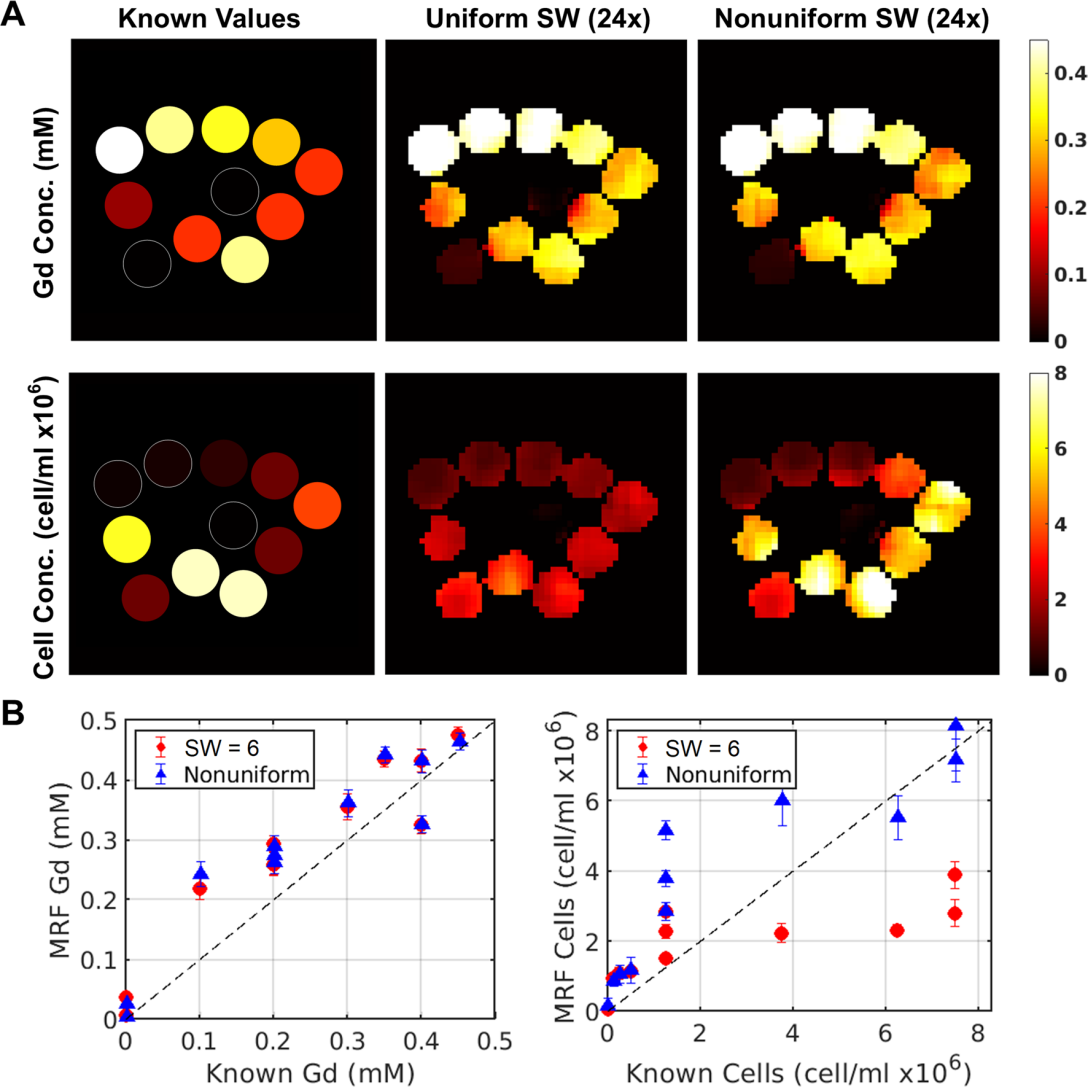
**A** Dual concentration maps generated using SW-MRF. **B** Comparison of the known values for each phantom, compared with the average over a 3 × 3 voxel ROI taken from SW = 6 MRF (circle) and nonuniform SW-MRF (triangle). SW = 3 concentration mapping is not included, as concentration maps were unable to be generated from the data

Figure 5a contains the results for concentration maps produced by MRF. Both SW = 6 and nonuniform SW-MRF perform Gd concentration mapping well, with results visually resembling the known concentration values. This is supported in Fig. 5b, where the inner 3 × 3 voxels of each phantom are compared to the known values of concentration, and are both found to have strong concordance correlation coefficients of 0.902 and 0.8874 for uniform and nonuniform SW-MRF respectively. SPIO-labelled cell concentrations are also mapped in Fig. 5a, and display a visible difference between the produced maps for both SW = 6 and nonuniform SW-MRF. Similar to *R*_2_* parameter mapping, uniform SW-MRF fails to accurately estimate the higher values of cell concentration, whilst nonuniform SW-MRF more closely represents the known values. Figure 5b shows that uniform SW-MRF fails to characterise phantoms above 2 million cells/mL, demonstrating the effect of inaccurate *R*_2_* mapping on cell concentration estimations. Nonuniform SW-MRF, whilst showing a tendency to slightly overestimate cells in the 2–4 million cells/mL range, provides a much more robust scaling into the higher values of cell concentrations. CCC values for SPIO-labelled cells support these findings, with values of 0.4666 and 0.8311 for uniform and nonuniform SW-MRF respectively.

## Discussion

In this study, we have demonstrated why care must be taken in the application of sliding window reconstruction to MRF, and shown that introducing a nonuniform sliding window length in the *T*_2_* sensitive regions of an MRF sequence can enable dual contrast quantification within an acceptable in vivo timeframe on single-channel preclinical systems.

The parameter validation results demonstrate that the nonuniform SW-MRF modifications improve the results for all parameter maps, combining the *T*_1_ and *T*_2_ accuracy of larger sliding window lengths, and the *T*_2_* sensitivity of shorter window lengths. This is visually apparent from the parameter maps, where SW = 6 clearly lacks *R*_2_ and *R*_2_* accuracy when compared to the known values, but offers improved robustness to the undersampling noise seen with SW = 3, with nonuniform MRF providing a balance between. For SW = 3, the shorter window length is unable to overcome the high undersampling factor noise, but is still able to provide *R*_2_* sensitivity, most likely due to the prominence of signal decay based on *R*_2_* dephasing. Conversely, SW = 6 underestimates either *R*_2_, *R*_2_*, or both, suggesting that longer window length uniform SW-MRF cannot accurately capture *R*_2_ and *R*_2_* dynamics, most likely due to the window length ‘smearing’ the *R*_2_* signal effects over more imaging frames. As higher *R*_2_* are typically present in studies involving iron-loaded cells, this highlights the need for optimisations such as the one proposed in this work.

The non-iterative nature of a SW acceleration approach means that no prior information is required for application, and that post-processing time is barely affected. This study shows that SW-MRF can enable a 24× increase in acquisition speed with minimal effect on data fidelity, whilst also leaving *T*_2_* sensitivity intact. The scan time of fully sampled MRF data using our current protocol is around 28 min without SW. This may be accelerated via 4× undersampling to ~400 s without compromising parameter map quality for either uniform or nonuniform MRF, but in this work we have demonstrated that nonuniform SW-MRF can further reduce this value to 80 s per slice.

CCC values for uniform and nonuniform SW-MRF can be found in Table 2(a). For every parameter, the nonuniform SW-MRF was found to consistently outperform both SW = 6 and SW = 3 MRF, with *R*_2_ and *R*_2_* showing increases from 0.2723 to 0.8837 and from 0.3482 to 0.8776 respectively when compared to SW = 6, and all values increasing when comparing nonuniform SW MRF to SW = 3. The only CCC which was not dramatically increased is SW = 6 and nonuniform *R*_1_ parameter maps, which is due to SW = 6 already performing well for *R*_1_ parameter measurement. We suspect that *R*_1_ sensitivity is maintained for longer window lengths due to the effects occurring over a longer timescale than *R*_2_ or *R*_2_*, lessening the effect of combining imaging frames. Overall, these results demonstrate nonuniform SW-MRF’s ability to strike a balance between the two uniform regimes. Wilcoxon signed-rank test results support this conclusion, showing that whilst SW = 6 *R*_2_* and SW = 3 *R*_1_/*R*_2_ averages differ significantly from the gold standard results, nonuniform SW does not.

Whilst a previous study on SW-MRF [18] showed that uniform SW enables accelerating MRF with accurate *R*_1_ and *R*_2_ measurements, this study highlights important cases in which additional care may be required. First, due to the way all parameters are dependent on each other in the generated dictionary and its contained fingerprints, inaccuracies in one parameter permeate into all others. It is likely that the errors seen in this study for *R*_2_ for the uniform SW = 6 MRF should be taken not as a demonstration of how poorly uniform MRF performs for these parameters, but rather how the presence of *R*_2_* contrast damages uniform SW’s ability to accurately accelerate MRF in general. An example of this can be seen with phantoms 7 and 11 in Fig. 3. Phantom 7 contains no SPIO-labelled cells, whereas phantom 11 contains the highest concentration, leading to a much higher *R*_2_*. Here we see that uniform SW cannot accurately measure *R*_2_* in phantom 11, leading to underestimates in *R*_2_, but has no problem measuring the *R*_2_ without the presence of a *R*_2_* contrast agent. Nonuniform SW can more accurately measure *R*_2_* in phantom 11, and as a result, *R*_2_ measurements are restored. Secondly, this study focuses on utilisation of highly undersampled MRF on a single-channel preclinical system, as well as imaging in the presence of iron. Thus, SNR is limited, which most likely accounts for SW = 3 MRF being unable to provide accurate *R*_1_ and *R*_2_ parameter maps.

Concentration mapping data show the clearest support for nonuniform over uniform SW-MRF, with SW = 6 MRF failing to capture the range of cell concentrations within the phantoms. Indeed, if not for the strong *r*_1_ relaxivity of the gadolinium agent, uniform SW-MRF would have most likely struggled to quantify both agents in the presence of *T*_2_*. A different agent such as manganese would be less reliant on *R*_1_ mapping for concentration calculations and would likely be more challenging to quantify alongside SPIO. Nonuniform SW-MRF, however, was able to provide accurate dual contrast parameter maps even at 24× undersampling. SW = 3 MRF concentration mapping performed so poorly due to noise that we were unable to produce concentration maps of any value.

Whilst nonuniform dual contrast SW-MRF does have a tendency to overestimate concentration values within the range of 2–6 million cells/mL, it still greatly outperforms SW = 6 uniform MRF over the entire range of cell concentrations mapped, having nearly double the CCC value. One source of error may be the phantoms themselves, in which accurate concentration values are difficult to ascertain, with iron cell loading varying anywhere from 3 to 5 pg/cell even within homogeneous cohorts. There may also be non-homogeneous distributions of cells within phantoms despite efforts to homogenise them. Therefore, there is inherent error in the *x*-axis of Fig. 5b which cannot be quantified. For this reason, the individual points are less valuable than the trend showing that measurement of cell concentration is applicable over a large range, with a well-defined minimum level of detection. B1 mapping corrections were not applied to any of the MRF sequences performed, which has been previously shown to directly impact the quantification errors in parameter mapping [23]. We suspect that adding B1 mapping corrections to the MRF workflow will therefore lead to increased accuracy for future SPIO labelling studies. Similarly, the EPG model used for this work does not employ a slice selective EPG (ssEPG) dictionary, which would allow for greater accuracy in modelling both slice-excitation and imperfect spoiling gradients [24]. However, we expect the lack of ssEPG and and B1 corrections to affect both nonuniform and uniform SW MRF equally, and therefore would not affect the comparison between the two. Another potential source of error is using a variation in TR to drive the non-steady state of the MRF sequence. Future optimisations for nonuniform SW MRF could include minimising the TR variation to provide a minimum amount of incorrect phase accrual, as well as employing an ssEPG and B1 mapping to improve quantification accuracy.

For this study, emphasis was placed on cell concentrations in the < 1 million cells/mL range, to provide greater characterisation of the minimum cell concentrations detectible, but future studies may benefit from further probing the 2–6 million cells/mL region. Whilst slightly overestimated, this study demonstrated detection of cell concentrations as low as 125,000 cells/mL, which is lower than cell densities seen in some previous in vivo studies [6].

It is important to note that for the most accurate parameter maps, MRF images would be required both prior to and after the introduction of contrast agents. Whilst the intended use for dual contrast SW-MRF involves in vivo applications, which would allow for such data to be collected, for in vitro data, we must rely on a simulacrum of pre-contrast data in the form of a phantom with zero contrast. As such, we see variation within each phantom, particularly in phantoms 6 and 11, but this is not something we expect to be a factor in the future in vivo studies.

In this study, we used a fixed ratio of 2 for the sliding window length between the *T*_2_* sensitive (*W* = 3) and non-*T*_2_* sensitive (*W* = 6) areas of the MRF sequence. This value was empirically chosen in an attempt to compromise between preliminary SW = 3 and SW = 6 results, in the hope of restoring *T*_2_* sensitivity. Whilst it is beyond the scope of the current study, further investigation into the effect of window ratio on parameter accuracy could further optimise nonuniform SW-MRF.

## Conclusions

In this study, the sliding window reconstruction strategy for MRF has been expanded to include a novel nonuniform window length. Whilst the existing SW methodology provided a means to improve the SNR and reduce undersampling artefacts in MRF data, this has proven insufficient for use on single-channel preclinical systems, compromising parameter accuracy. By lowering the window length during the *T*_2_* sensitive areas of the MRF sequence, it has been demonstrated the nonuniform SW-MRF increases the accuracy of all parameters in the presence of *T*_2_* contrast in vitro, leading to more accurate concentration maps for contrast agents. This acceleration optimisation is critical for any preclinical application of MRF which hopes to use SW as an acceleration technique to measure samples with a *T*_2_* contrast, such as time-sensitive in vivo experiment in which iron is used to label cells of interest.
